# Influence of the Lower Jaw Position on the Running Pattern

**DOI:** 10.1371/journal.pone.0135712

**Published:** 2015-08-13

**Authors:** Christian Maurer, Felix Stief, Alexander Jonas, Andrej Kovac, David Alexander Groneberg, Andrea Meurer, Daniela Ohlendorf

**Affiliations:** 1 Move functional, Salzburg, Austria; 2 Orthopaedic University Hospital Friedrichsheim gGmbH, Frankfurt/Main, Frankfurt am Main, Germany; 3 Department of Movement and Exercise Science, Institute of Sport Science, Goethe-University Frankfurt/Main, Frankfurt am Main, Germany; 4 Institute of Occupational Medicine, Social Medicine and Environmental Medicine, Goethe University Frankfurt/Main, Frankfurt am Main, Germany; New York University College of Dentistry, UNITED STATES

## Abstract

**Introduction:**

The effects of manipulated dental occlusion on body posture has been investigated quite often and discussed controversially in the literature. Far less attention has been paid to the influence of dental occlusion position on human movement. If human movement was analysed, it was mostly while walking and not while running. This study was therefore designed to identify the effect of lower jaw positions on running behaviour according to different dental occlusion positions.

**Methods:**

Twenty healthy young recreational runners (mean age = 33.9±5.8 years) participated in this study. Kinematic data were collected using an eight-camera Vicon motion capture system (VICON Motion Systems, Oxford, UK). Subjects were consecutively prepared with four different dental occlusion conditions in random order and performed five running trials per test condition on a level walkway with their preferred running shoes. Vector based pattern recognition methods, in particular cluster analysis and support vector machines (SVM) were used for movement pattern identification.

**Results:**

Subjects exhibited unique movement patterns leading to 18 clusters for the 20 subjects. No overall classification of the splint condition could be observed. Within individual subjects different running patterns could be identified for the four splint conditions. The splint conditions lead to a more symmetrical running pattern than the control condition.

**Discussion:**

The influence of an occlusal splint on running pattern can be confirmed in this study. Wearing a splint increases the symmetry of the running pattern. A more symmetrical running pattern might help to reduce the risk of injuries or help in performance. The change of the movement pattern between the neutral condition and any of the three splint conditions was significant within subjects but not across subjects. Therefore the dental splint has a measureable influence on the running pattern of subjects, however subjects individuality has to be considered when choosing the optimal splint condition for a specific subject.

## Introduction

Recent debates suggest an anatomical and functional correlation between the whole body posture and the tempo-mandibular system (TMS) [1–4]. However, the interaction between occlusion, TMS and posture is discussed controversially [2, 3, 5–9]. Some authors could not found an interaction between the occlusion, the TMS and posture [7, 10–13]. However, other authors reported a functional interdependence between the TMS and body posture [1, 6, 8, 9, 14–20]. The interaction effects were found for the healthy TMS [6, 9–13] as well as for patients with tempo-mandibular disorders (TMD) [7, 8]. In all studies the dental occlusion position was manipulated. It has been shown that small interventions at the jaw have significant effects on posture and gait stability [21–23]. To provoke different dental occlusion positions altered strategies are utilised, like cotton rolls, silicon wafers, tin foils or dental splints [1, 19, 21, 23, 24].

The current understanding is that the TMS and the neuromuscular system of the whole body are connected via the central nervous system [23, 25]. In animal studies a link between neurones of the cranio-mandibular system and structures of the central nervous system could be found [26–28]. A speed dependent reflex response in posture could be observed while the jaw position was changed [29–32]. Therefore it is speculated that the afferent pathways from the TMS are connected with efferent neurones that affect body posture [33].

In addition to the neuronal connection of TMS and body posture it was speculated that the TMS and the muscles of the body are connected with the fascia system through muscle and fascia chains, respectively [33]. To date no direct prove of either the neuronal or fascial connection of the TMS and the musculoskeletal system is available. However the indirect evidence suggests a moderate influence of the jaw position on body posture [1, 6–12, 16–18, 20–24, 33, 34].

The effects of a manipulated dental occlusion on body posture have been investigated quite often in the literature [1, 9, 19, 20, 22–24]. The effects of the dental occlusion position on human movement have been paid less attention. If human movement was analysed, it was mostly while walking [6, 18, 23, 35] and not while running. The influence of the occlusion on global gait parameters like gait velocity and gait cycle, as well as the influence on kinetic variables such as plantar pressure distribution could be demonstrated [6, 18, 21, 35].

The question remains, whether the dental occlusion can affect human motion even during sport activities, where the range of the movement is much larger than during standing or walking. To identify small changes of such movement patterns, sophisticated analysis techniques have to be applied to the data. Vector based pattern recognition methods such as cluster analysis, support vector machines (SVM) and principal component analysis (PCA) have become useful tools in movement pattern identification [36–40]. They have been successfully used to identify small changes of the movement pattern introduced for instance with different footwear [41] or to characterise the gait pattern of healthy humans [42]. In order to differentiate random fluctuations of the gait pattern from systematic differences between two conditions the variability of the gait pattern can be used [43].

With the above mentioned mathematical tools the question, whether dental occlusion affects the running pattern can be addressed. The main aim of the present study was to assess the effect of a changed dental bite position on the running symmetry. The dental change was introduced by lower jaw splints. Three different splints were tested during this examination and the three conditions were compared with the condition where the lower jaw was at rest. The hypotheses to be tested were:

Hypothesis 1: Lower jaw splints change the movement pattern in a consistent manner during running.

Hypothesis 2: A splint induced change of the dental occlusion increases the symmetry of the running pattern.

## Materials and Methods

### Subjects

Twenty healthy young recreational runners (5f/15m) participated in this prospective study. The mean and standard deviation age was 33.9 ± 5.8 years. Only subjects meeting all inclusion criteria were included in this study. Inclusion criteria were assessed with a questionnaire on the recruitment day [44]. Inclusion criteria were:

- weekly mileage above 25km, average running speed above 2.7 m/s, running duration per session above 50 minutes [45];
- no acute pain in the musculoskeletal system that prevents or affects the execution of ordinary activities;
- no abnormalities, traumas or surgeries of the musculoskeletal system as well as in the upper or lower jaw within the last two years;
- no current orthodontic or orthopaedic treatment;
- no locking or abnormal stiffness of the jaw;
- no acute infection, drugs or any vestibular or somatosensory disorder.

The rights of these subjects were protected and they were thoroughly familiarised with the study design before giving written informed consent to participate in this study, as approved by the local medical ethics committee of the medical faculty of the Goethe-University (Nr. 331/11) and in accordance with the 1964 Helsinki Declaration and its later amendments.

### Experimental protocol

#### Occlusion conditions

Four different dental occlusion conditions were investigated: 1st a relaxation splint in the centric condylar position (referred as centric) [5]; 2nd a dental power splint in the myocentric condylar position (referred as DPS) [22]; 3rd a maximum intercuspidation splint (referred as Max); 4th the habitual occlusion position at rest (referred as Neutral) [5]. The first three occlusion conditions were accomplished with mandibular splints positioned in the lower jaw.

**Fig 1:**
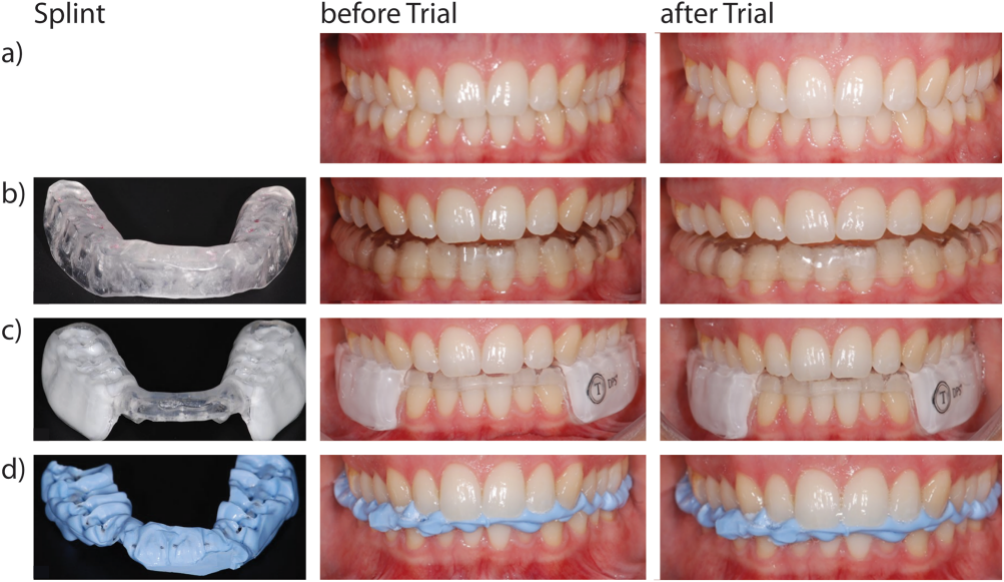
Illustration of the splint conditions. a) Neutral b) Centric, c) DPS, d) Max. A detailed description of the splints is provided in the text.

All three splints were individually manufactured. For the centric splint condition the lower jaw was placed in the habitual occlusion position (the habitual occlusion is defined as the static occlusion a person usually assumes which furthermore determines the habitual condylar position [5]), for which a face-bow transfer and a subsequent cephalic articulation of the respective maxillary model to the articulator (Protar Evo 9; KaVo Dental, Bieberach, Germany) is necessary. Furthermore, a registration template of light-curing synthetic was produced for the centric registration at a laboratory. A frontal stop in the sense of a Jig (Bite Compound, GC Germany, Bad Homburg, Germany) was applied to the registration template and stretching exercises of the whole body and the mandible were additionally performed to resolve the usual final bite position. With the aid of a bite registration material (DMG, Hamburg / Germany LuxaBite) the bite was fixed on the template in the mouth, so that the lower jaw gypsum model could be incorporated into an articulator with the help of this template. The articulator was programmed in accordance with the data from a previously conducted axiography (Arcus Digma; KaVo Dental, Bieberach / Germany). On the surface of the deep drawn film of the lower jaw model acrylate (Durasplint; Scheu Dental, Iserlohn / Germany) was applied, so that the splint could be adapted precisely to the maxillary teeth in the articulator (Fig 1).

For the DPS splint the most favourable myocentric bite position had to be found. This myocentric bite position is the occlusion position where the mandible has a balanced isotonic muscle contraction [22]. The position can be found with transcutaneous nerve stimulation (TENS) [46]. Therefore subjects obtained a one hour TENS session, where the electrodes were placed on the left and right M. masseter and M. temporalis. The state of the craniomandibular system was recorded with a mandibular scanner (K7, Myotronics, Seattle/USA). The lower jaw splints were again manufactured from the registration template after a certain procedure which is described elsewhere [22].

Finally the Max splint fixes the habitually occupied relation of the upper jaw to the lower jaw. The patient bites on a registration template in maximum intercuspidal position. In the Max splint condition the maximum number of multiple contacts between the teeth of the upper and lower jaw exists (Fig 1D).

#### Running setup and recording

The standardised Plug-in-Gait marker set was applied to determine joint centres and kinematic data. Reflective markers were placed by the same experienced investigator on well-defined anatomical points as described in a previous investigation [15] to define the pelvis, thigh, shank and foot segments. Additional markers on the upper body were attached to record trunk, neck and head movements according to an enlarged model, which is part of the Vicon software (VICON Motion Systems, Oxford, UK). Reflective markers were recorded using the MX T10 Vicon motion capture system (VICON Motion Systems, Oxford, UK), operating at a sampling rate of 200 Hz and a resolution of 1 megapixel. The level walkway was 15 meters long and viewed by eight infrared cameras. Two AMTI force plates (Advanced Mechanical Technology, Inc., Watertown, MA, USA), synchronised with the motion capture system were situated at the mid-point of the walkway to define the instants of heel strike (first ground contact) and toe-off (last ground contact). Subjects were prepared with the four occlusion conditions in random order. Subjects performed five running trials on the level walkway with their preferred running shoes on the basis of complete marker trajectories and a clear foot-forceplate-contact per test condition. Before every test condition subjects were asked to make a warm up run for five minutes on a treadmill (Daum Electronic GmbH, Fürth, Germany) at a speed of 2.7 m/s to become familiar with the new occlusion condition. All subjects got the instruction to bite on the splint with moderate force during the measurement time and while running.

Between the test conditions subjects were given a five minute rest where they were asked to move around and perform light stretching exercises [47]. Recorded data are proved in the supporting information (S1–S7 Files).

#### Data preparation

After each acquisition session, 3D marker trajectories were reconstructed and missing frames were handled with a fill-gap procedure using the Vicon-Nexus software version 1.7.1 (VICON Motion Systems, Oxford, UK). The kinematic data were spline smoothed with a Woltring filter [48].

Kinematic data were analysed for the whole running cycle of the right and left side. Intersegmental joint angles were calculated for the ankle, knee, hip, spine, and neck. In addition, the segment angles for the foot, pelvis, thorax, and head were calculated with reference to the global coordinate system of the walkway.

Only sagittal plane angles were used for further calculations, because frontal and transverse plane angles were deemed to have an increased measurement error. Kinematic data were time normalized and interpolated at 201 equidistant time points for one running cycle. All data were combined in one input matrix, with trials from all subjects, conditions and sides in the rows and the time dependent angles of the foot, ankle, knee, hip, pelvis, spine, thorax, neck and head connected in the columns resulting in 682 rows and 1809 columns. The mean values over all subjects were subtracted:

$${M^{\prime }}_{j}(k)=M_{j}(k)-\bar{M(k)},$$

with j being the index of the trial, and k the index of the variable set. A variable set was defined as one angle over the 201 time points. This keeps the time dependent variation of the angles. $\bar{M(k)}$ and *SD*{*M*(*k*)} are the mean and standard deviation over the trials, conditions and subjects, respectively. The resulting input matrix *M*′ had zero mean and standard deviation of one for every variable set.

#### Data mining

**Fig 2:**
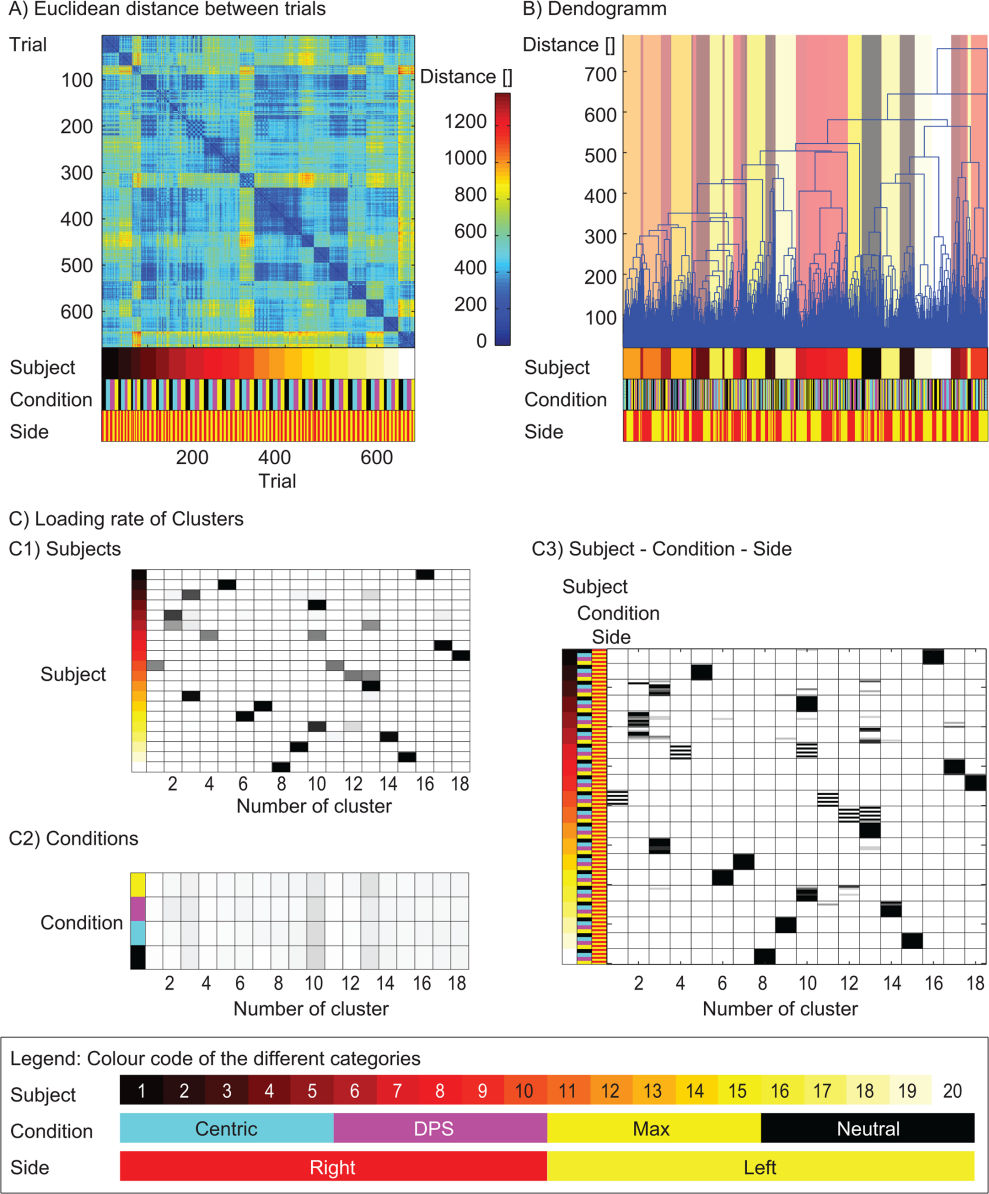
Cluster analysis. All trials of all conditions were subjected to a cluster analysis. The colour code for the three different categories is plotted in the legend. A: Distance matrix between each trial. Trials are plotted in the x and y direction. The colour of the image gives the distance between two trials. The contribution of the trials to subject, condition or side is colour coded in the legend underneath the image. B: Dendogram to visualize the similar characteristics of different trials. The trials on the x-axis are organized depending on the distance between them. The distance on the y axis is a measure for the number of steps required to combine sets of trials. C Loading rate of subjects (C1), conditions (C2), and subject, condition and side (C3). The rate is a value between 0 and 1 depending if no trial is loaded in a cluster or all trials are loaded in the same cluster.

A first general picture of the data was drawn with a cluster analysis [36, 37, 39, 49]. The input matrix M was subjected to a K-mean cluster analysis with squared Euclidean distance (matlab R2013a). The best number of clusters was estimated with a similarity measure, where the function silhouette was used (matlab R2013a). This function estimates the distances of trials within a cluster and compares these distances to the distances to the trials of the other clusters. A value close to one states that all trials of one cluster are close to each other but very distant to the trials of all the other clusters. The number of clusters with the highest silhouette value was used for further analysis [50]. The difference between every trial was plotted (Fig 2A) and a dendogram showing the number of steps required to connect two trials was plotted (Fig 2B) [49]. Furthermore the loading rate of trials combined in every cluster was calculated. This was done (1) for all trials per subject (Fig 2C1), (2) for all trials per condition (Fig 2C2) and (3) for all trials per subject per condition per side (Fig 2C3). The loading rate was the number of trials of one subject or one condition in one cluster divided by the total number of trials for that subject or that condition.

**Fig 3:**
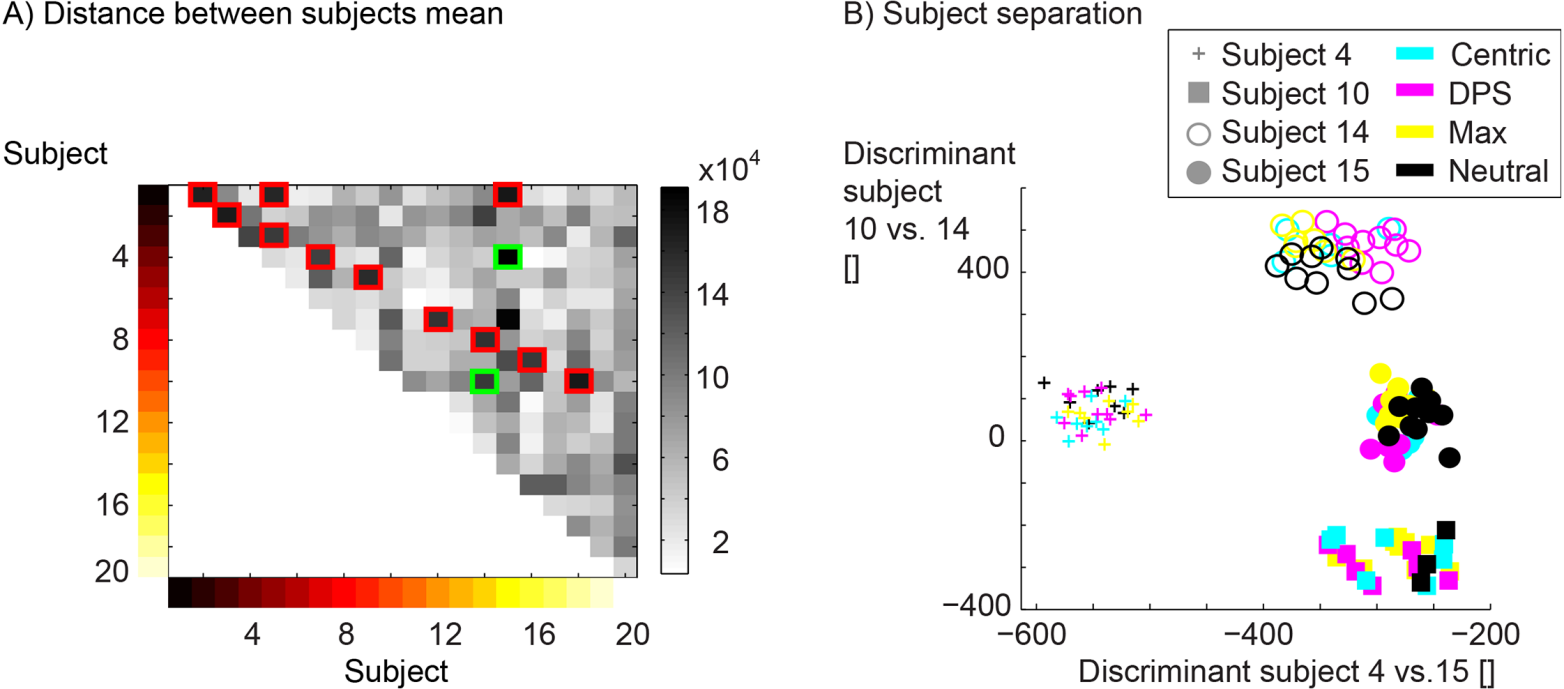
Separation of subjects. A: Distance matrix between subjects. The grey values indicate the distance between subjects. The red squares indicate the 11 comparisons that were not significantly classified. For the 4 subjects indicated by the green squares (two subjects are combined in every square) the projection on the discriminant was calculated and plotted in the graph B. B: Trials of the same subject were indicated by subject specific colour of the outer circle. The colours of the 4 subjects were green (10), red (14), magenta (4) and blue (15). The trials of the same condition were colour coded in the inner circle. The colour for the condition is given in the legend.

Based on the cluster analysis subject and condition specific differences were explored. This was done with a SVM with a linear kernel [39, 42]. A leave one out method was used to calculate the classification rate. The SVM was applied for three different categories. 1^st^ category was built from all trials of two subjects. All combination of subjects were compared in order to determine if subjects moved in a subject specific way (Fig 3A). 2^nd^ category was based on the three different splint conditions against the normal condition across all subjects. This was performed in order to find if the different conditions have a subject independent influence to the movement pattern. 3^rd^ category was the condition dependent analysis within each subject. This was done to see if the condition influences the movement pattern on a subject specific level.

**Fig 4:**
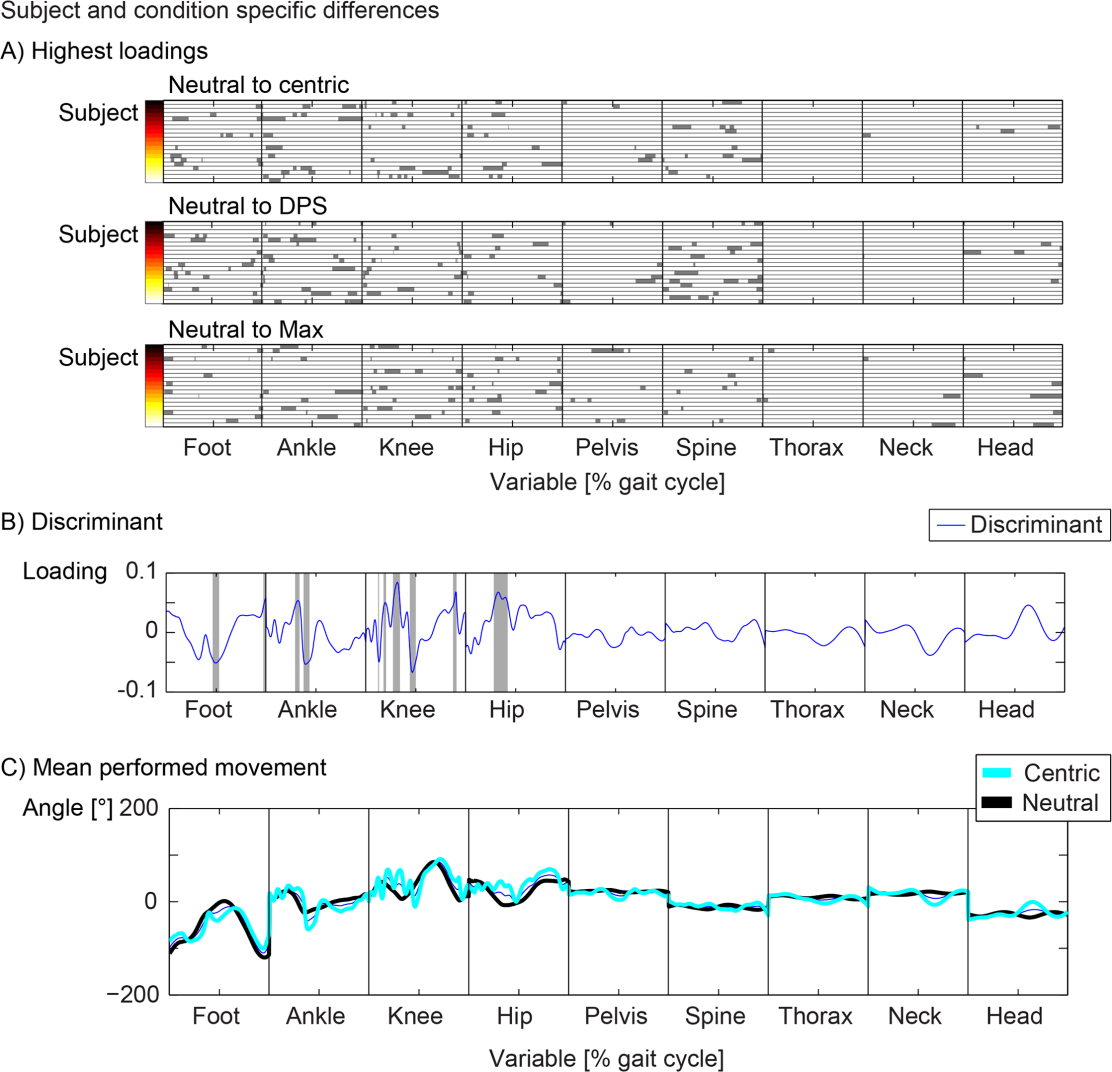
Subject specific differences of the movement pattern between the conditions. A: Main contributions to the discriminant between different conditions and subjects. B: One example: Discriminant (blue line) between neutral and centric for subject 4, the highest contributions to the classification of the two conditions are indicated by the grey area. C: Movement pattern of the neutral (black) and centric (cyan) condition.

If a significant classification could be calculated, the discriminant separating the two groups maximally was calculated. Variables that have a high loading on the discriminant are variables that allow the classification of the two groups and are therefore important variables for the separation. These variables were found by calculating the standard deviation across all loadings of the discriminant and finding all variables that have a higher absolute value than two times the standard deviation. The discriminants for all conditions and subjects were plotted and variables exceeding two times standard deviation were highlighted (Fig 4A). All calculations within the framework of the SVM are bijective. Therefore it is possible to calculate back to the actual movement values (Fig 4C). The difference in the movement between two conditions for one subject was presented as one example.

**Fig 5:**
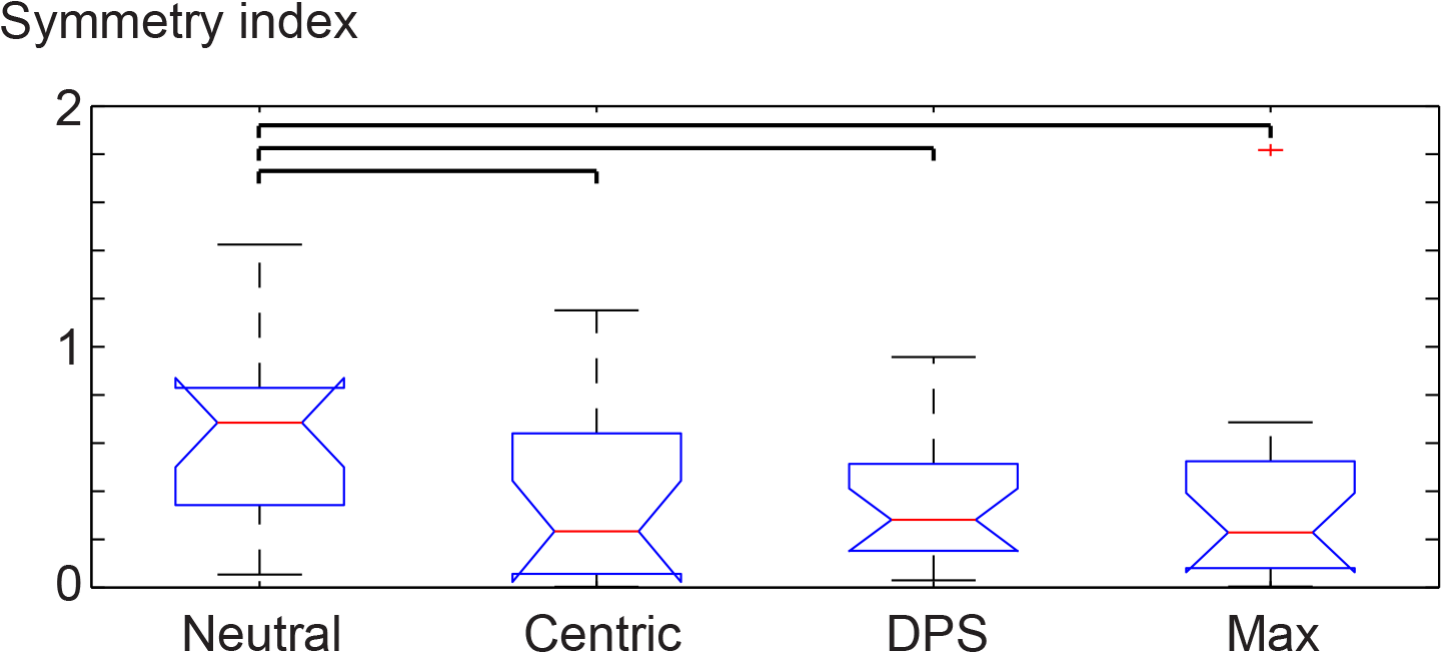
Symmetry index of the different conditions. The bars on the top indicate the significant differences between the four groups. The three interventions are more symmetric than the neutral condition, but no intervention is more symmetric than another intervention.

Changes in the movement symmetry were assessed by comparing the mean over all trials from the left side to the mean over all trials from the right side [51, 52]. This was done for each splint condition separately as well as for the neutral position (rest position). The mean difference was weighted by the pooled standard deviation in order to account for the variability of the movement. Therefore, the mean waveform was calculated, the left side was subtracted from the right side, each time point was subsequently divided by the pooled standard deviation. The remaining waveform was summed and the absolute value was taken to get a single symmetry value per subject. This symmetry value is normalized to the standard deviation and therefore dimensionless (Fig 5).

#### Statistics

For testing the running speed an ANOVA was used because of the normal distribution of the data (Kolmogorov-Smirnov-Lilliefors-Test) followed by a paired t-test and Bonferroni-Holm correction.

To estimate the significance level for the classification rate a binominal distribution with probability of 0.5 and α set to 0.05 was used to determine the number of correct classified trials to achieve a significant classification rate. For not normal distributed data a Kruskal-Wallis rank sum test was used to determine significance in multiple group comparisons. The α was set to 0.05.

For the gender analysis a Friedman test and a followed subgroup analysis were used. This analysis can be regarded as a secondary test. Therefore, no alpha correction was used. For the subgroup analysis the Wilcoxon-Mann-Whitney U-test was used. Afterwards the results were related to different effect sizes to Rosenthal. An effect size of 0.1 was determined as a low effect size, 0.3 as a medium effect size and 0.5 as a large effect size.

## Results

### Running Speed

No difference in the running speed between the four conditions could be found (p = 1.00).

### Movement pattern classification

18 clusters were deemed to be the optimal number of clusters. The distance matrix showed a clear pattern, where subjects seemed to be close together (Fig 2A). The distance between different conditions and different sides seemed to be more variable. A similar picture can be seen when plotting the number of steps required to connect different trials (Fig 2B). The loading rate of trials within the same cluster was plotted for the subject group, the condition group and the most detailed group of subject, condition and side (Fig 2C1, 2C2 and 2C3). Eight out of twenty subjects were fully combined in one cluster. Six subjects had a loading rate between 0.9 and 1, two between 0.8 and 0.9 and the remaining four subjects had a loading rate lower than 0.8. The maximal loading rate for the condition dependent clustering was 0.18 with a median value of 0.048. In total 160 categories where measured if one splits the trials into subjects, condition and side. 134 out of these 160 categories had a loading rate of 1 (83.7%).

### Subject specific differences

With 20 subjects 190 possible pairwise combinations exist. For 179 of these combinations the movement pattern of the subjects could be classified (Fig 3A). The total number of comparisons was 190. The distance between conditions within a subject and the distance between subjects was plotted for four subjects as example (Fig 3B).

### Condition dependent differences

The classification rate between the three interventions and the neutral condition across all subjects was: r_Class_ = 0.53 (neutral to the centric), r_Class_ = 0.52 (neutral to DPS), r_Class_ = 0.51 (neutral to Max).

Within individual subjects the splint conditions showed distinct movement patterns. The mean classification rate for the condition and subject specific separation was r_Class_ = 0.81 (neutral to the centric), r_Class_ = 0.84 (neutral to DPS), r_Class_ = 0.78 (neutral to Max) (Table 1).

**Table 1:** Subject specific classification of different conditions.

Subject specific classification rate	1	2	3	4	5	6	7	8	9	10	11	12	13	14	15	16	17	18	19	20
Neutral to centric	**0.89**	**0.79**	0.68	**0.79**	**0.95**	**1.00**	**0.75**	**0.95**	**0.75**	0.45	0.60	**0.90**	0.65	**0.80**	**0.70**	**0.90**	**0.90**	**0.95**	**0.80**	**1.00**
Neutral to DPS	**0.90**	0.50	0.50	**0.95**	**0.95**	**0.80**	**1.00**	**0.95**	**0.90**	**0.75**	**0.80**	**0.90**	**0.90**	**0.95**	**0.75**	**0.70**	**1.00**	**0.80**	**0.85**	**0.95**
Neutral to Max	**0.89**	**0.83**	0.67	**0.83**	0.67	0.61	**0.75**	**0.85**	0.65	**0.80**	**0.70**	**0.70**	**0.95**	**0.75**	0.65	**0.85**	**1.00**	**0.85**	**0.80**	**0.85**

The classification rate within each subject is mentioned for the three comparisons: Neutral to centric, neutral to DPS and neutral to Max. Significant classification rates are highlighted in bold.

The main differences between the three splint conditions and the neutral condition were found at the ankle, knee and spine. Hardly any contributions were detected for the thorax and neck (Fig 4A). These differences were highlighted with a grey zone. The differences change between subjects and conditions. For one example, the discriminant and the highlighted area of main differences were plotted (Fig 4B). The back calculated values for the time dependent variables indicated the differences between the two conditions in angles (Fig 4C). It is important to note that the mean line of the movement showed a difference between the two conditions but the loading of the same variable on the discriminant is close to zero. The variability of this movement is high and therefore this time point of the variable does not contribute to the separation of the two conditions.

### Condition dependent symmetry

The median of the symmetry between left and right leg for the four conditions was 0.69, 0.23, 0.28 and 0.23 for the neutral, centric, DPS and max condition, respectively. The neutral condition was significantly more asymmetric than any of the three splint conditions Χ^2^ (3,69) = 7.86 (p = 0.049) (Fig 5). No difference can be found between the three conditions.

However, the result was dominated by the female subgroup. A Friedman test on the two subgroups resulted in different relationships for the two gender groups. For the male subgroup the symmetry was not significant: X2 (3,11) = 2.8 (p = 0.43), while for the female subgroup a significant result was found X2 (3,5) = 9.96 (p = 0.02). The neutral condition showed a less symmetric pattern than any of the three splint conditions, again no difference between the splint conditions could be found.

After the Friedman-test the Wilcoxon-Mann-Whitney U-test showed significance in the Max of p ≤ 0.04 with a large effect size of 0.5. The other occlusion comparisons were not significant. The effect sizes of the non-significant comparisons were 0.41 at neutral, 0.21 to centric and 0.19 to DPS.

## Discussion

The rationale of the present study was to assess the influence of the lower jaw position on the running pattern. Since the running speed was not different between all trails and all subjects, further analysis of the data could be carried out.

Firstly, the optimal number of clusters was close to the number of subjects included in the study. In general, the different trials within one subject were closer to each other than the trials between conditions across subjects (Fig 2B). The loading rate for the three categories (1) subjects, (2) conditions and (3) subject-condition-side confirmed this impression. While the loading rate for the category subject was above 0.8 for 16 out of 20 subjects, the rate was below 0.18 for the category condition. This suggests that the changes between different conditions within one subject were smaller than the changes between different subjects which was confirmed by the subject specific classification. Consequently, the trials of one subject could be separated from the trials of another subject independent of the condition. The conditions however could not be separated independent of the subjects. Therefore, the hypothesis that the splint conditions lead to a unique movement pattern that is independent of subjects has to be rejected.

Within a subject, who showed a unique pattern, the splint conditions introduced a change of the movement pattern that was consistent throughout the measurement time. These changes were different for various subjects and conditions. Since these changes were small in relation to the movement differences between subjects, they were hard to detect (Fig 3B). Each group consisted of 20 subjects. The neutral condition could be significantly separated for 16 subjects for the centric, 18 subjects for the DPS and 15 subjects for the Max condition respectively (Table 1). Therefore within a subject the movement pattern of different conditions could be separated for the majority of subjects. The main differences between the three splint conditions and the neutral condition were found at the ankle, knee and spine. The influence of the splint was therefore even measured in distal regions. Why the effects of an occlusal splint can be seen in more distal structures, which are not primarily associated with the TMS has to be investigated in further studies. It might be speculated that the splint changes mainly the head position. Consistent changes in the head position were found for 2, 4, 5 subjects for the Zentrik, DPS and Max condition, respectively (Fig 4A). More subjects showed changes in the lower extremity and the spine. In summary the main effects of the splint conditions could be seen in the lower extremity and the head position only changed for some subjects in a consistent manner.

The comprehensive analysis approach allowed the identification of these small changes. Wearing the splint conditions reduced the difference between the left and the right movement pattern. This change towards a more symmetrical running pattern could be observed for all three splint conditions. In general there is not one condition that corrects the movement pattern to a more symmetrical movement pattern. The splint condition that induces the most symmetrical running pattern has to be found on an individual base. Nevertheless, hypothesis 2 can be verified. The observed change in asymmetry might arise from different sources introduced because of the splint conditions. Such sources could be a change in swallowing, breathing or the closure of the lips. However in the presented study such changes were not assessed. However the significant change in symmetry was dominated by the small subgroup of female subjects. In the subgroup analysis the significant Max condition showed a large effect size (0.5). The small effect sizes in DPS and centric suggests that the sexes have no relevant differences. In neutral although no significance exists, but the effect size is 0.41, so that it can be concluded that one would expect a significance in the gender comparison with increased sample size (insufficient statistical power). The reason for these different reactions between male and female subjects in the different occlusion positions should be analyzed in further studies.

An improvement in symmetry is often considered beneficial for injury prevention and performance [51, 52], and it could therefore be possible that the splint conditions introduce a beneficial change in terms of a more symmetrical movement pattern. However these changes of the movement pattern due to the splint condition were small, and the relevance of these changes could be questioned. The importance of the introduced changes was not assessed in this work and has to be investigated in further studies. Another study aiming for instance for the benefits with respect to pain, injury prevention or performance would be necessary to explore the relevance of the changes introduced by the splint conditions.

Although the influence of the splint condition is small it is important to note that the splint condition did change the movement pattern. Therefore, depending on the goal of the dental intervention a subject specific solution has to be found. For instance, while all conditions lead to a more symmetric running pattern, the splint conditions might lead to subject specific changes of the running pattern. This implies that the splint conditions introduce a subject specific change of the movement pattern and an individual assessment of the consequences of the splint conditions on the movement pattern has to be performed.

The individual change of the movement pattern by wearing a splint may cause primarily a shifting of the structures of the TMS. A splint-caused change of the mandibula position can decompress the jaw joint on both sides of the body which may lead to a relaxation of the jaw muscles. Furthermore the compression of the jaw joints can be avoided while biting. Imbalances of the TMS can be adjusted and an improved coordination of the structures of the TMS generate a more harmonious muscle behaviour in terms of an improved muscular balance. A harmonisation of the TMS structures may affect the whole movement apparatus through caudal muscle and fascia chains as described above.

A general change in movement pattern in athletes (field hockey and boxing) wearing a mouth guard could also be proven in other investigations [53, 54]. These mouth guards are produced in centric relation. The authors assumed that these changes in posture may affect the precision of the technical execution in hockey or boxing strokes. However, the difference between a mouth guard and a splint has to be taken into consideration. Beside the thickness of the dental appliance, the occlusion position in which the appliance is produced is of importance. Another fact that has to be taken into consideration is the splint position either in the lower or in the upper jaw. While the splint in this paper is positioned in the lower jaw, a mouth guard is normally positioned in the upper jaw. Consequently, these facts should not be neglected if both appliances were compared with each other. Although studies proved the influence of a mouth guard on the respiratory system [55, 56], it has not been investigated whether a dental splint can effect the same results as a mouth guard [57]. Here, it has to be taken into consideration that differences can be due to different vertical heights or a general difference in the thickness of the dental appliance [57]. Other studies have proved the effects of a mouth guard on postural control or performance but not on running pattern [58, 59].

The use of a maximal oxygen consumption measurement would be helpful in such studies. Whether there is a difference on the symmetry of running in athletes between wearing the appliances for the first time and wearing the appliance for longer period of time could not be answered. The authors acknowledge that 5 minutes for familiarisation of each occlusion condition is very short. Since other studies reported significances after such short times while walking this study protocol was set similarly [23, 29–32, 60]. A follow-up study is therefore necessary to investigate differences between a short and a long-term familiarization.

This study investigated only the acute effects of the splint conditions. The development of the kinematic pattern over several days and if changes of the splint conditions would be reversible have to be investigated in a future study. In addition, the actual bite force was not examined and it could well be that differences in the bite force between the different splint conditions are the reason for the changes in the kinematic pattern. Up to now no study has investigated the bite force during continuous biting on a splint and during specific movements. Currently no valid measurement devices exist in dentistry that can measure bite force during movement only during standing [61].

Subjects had in summary distinct movement patterns. This movement pattern changes by a small amount when the splint conditions were worn. The differences of the movement pattern between different subjects were much larger than the changes introduced by the splint conditions. The change of the movement pattern between the neutral condition and any of the three splint condition was significant. Subjects changed their movement pattern on an individual base. The splint conditions introduced a change in the movement pattern. Therefore, splint conditions could be used to change the movement pattern of subjects even during running. A more symmetrical running pattern might help to reduce the risk of injuries or help to increase performance.

## Supporting Information

S1 File The file was compressed with Winrar. The file size is 5MB. The data processed in this paper can be found in the Supporting Information. The matlab database.mat was split into seven files with winRar. The authors confirm that the files do not contain any patient-identifying information, and that publication will not breach any ethical or legal regulations.
(RAR)

S2 File The file was compressed with Winrar. The file size is 5MB.
(RAR)

S3 File The file was compressed with Winrar. The file size is 5MB.
(RAR)

S4 File The file was compressed with Winrar. The file size is 5MB.
(RAR)

S5 File The file was compressed with Winrar. The file size is 5MB.
(RAR)

S6 File The file was compressed with Winrar. The file size is 5MB.
(RAR)

S7 File The file was compressed with Winrar. The file size is 78KB.
(RAR)
